# Factors associated with in-hospital mortality from community-acquired pneumonia in Portugal: 2000–2014

**DOI:** 10.1186/s12890-019-1045-x

**Published:** 2020-01-21

**Authors:** Ezequiel Pessoa, Cristina Bárbara, Laura Viegas, Andreia Costa, Matilde Rosa, Paulo Nogueira

**Affiliations:** 1 Escola Superior de Enfermagem de Lisboa, Lisbon, Portugal; 2 Direção-Geral de Saúde, Lisbon, Portugal; 3 Instituto de Saúde Ambiental (ISAMB), Faculdade de Medicina, Universidade de Lisboa, Lisbon, Portugal; 4 Centro Hospitalar Universitário Lisboa Norte, Lisbon, Portugal; 5 Laboratório de Biomatemática, Faculdade de Medicina, Universidade de Lisboa, Lisbon, Portugal

**Keywords:** Community-acquired pneumonia, In-hospital mortality, Portugal

## Abstract

**Background:**

Community-acquired pneumonia (CAP) is one of the leading causes of morbidity and mortality worldwide, often leading to hospital admissions. In Portugal, the factors associated with in-hospital mortality due to CAP are not fully documented. The aim of this study was to characterize the trends of CAP hospitalization in all age groups and the factors associated with their mortality between 2000 and 2014.

**Methods:**

We conducted a cross-sectional study using CAP hospitalization data in all age groups, in Portugal Mainland. Logistic regression was used to identify the factors associated with in-hospital mortality.

**Results:**

Between 2001 and 2011, CAP hospitalization rate increased from 2.8 to 4.3 per 1000 population. Hospitalization rates were higher in the extreme ages ( ≤ 4 and  ≥ 75 years). However, a decrease in the hospitalization rate and its mortality was observed, in the younger ages.

A total of 548,699 hospitalization CAP episodes, between 2000 and 2014, were analyzed, with male (56.2%) and elderly ≥65 years (91.7%) predominance, resulting in 101,740 deaths (18.5%). Men had a significantly lower mean age (64.3 ± 26.4 years versus 67.9 ± 27.5 years; *p* < 0.001). During the studied 15 years, there was an increase of 45.2% in the number of annual hospitalizations, concomitant with the admission increase of individuals aged over 75 years. Since 2012 a decrease in hospitalizations and associated deaths were detected.

The increase in age represented a progressive and significant rise in the probability of death, except for the age group 1–4 years. The age group ≥85 years old (Adjusted OR = 124.256; 95%CI: 97.838–157.807) and males (Adjusted OR = 1.261; 95%CI: 1.243–1.280) were significantly associated with death risk for CAP hospitalization. After 2010, this risk decreased (Adjusted OR = 0.961; 95%CI: 0.940–0.982). The main factors affecting mortality were age, sex, unemployment rate, number of performed procedures and admission quinquennia.

**Conclusions:**

Despite a trend of decrease in CAP hospitalizations and associated death since 2012, the numbers of in-hospital mortality showed, in the 15 years under analysis, an overall increase over time, mainly associated with age, in particular very old people ( ≥ 75 years), males and a higher parish unemployment rate. Therefore, the implementation of CAP preventive measures should be reinforced in these vulnerable groups.

## Introduction

### Background

Community-acquired pneumonia (CAP) is one of the main causes of morbidity and mortality worldwide [1, 2]. Globally, pneumonia, together with other lower respiratory tract infections are the leading cause of death from infectious disease causing 3.0 million deaths worldwide and being the fourth cause of death from all causes in 2016 [2]. CAP mainly affects the elderly in developed countries and children in developing countries [2]. Also, in the European Union (EU) pneumonia remains the most frequent cause of death from infection and was responsible for nearly 140,000 deaths in 2015, accounting for over 30% of all respiratory disease mortality [3]. There are large variations in mortality rates across EU countries: Portugal, the Slovak Republic and the United Kingdom have the highest rates of pneumonia mortality [3]. Despite this epidemiological information, it is difficult to determine the real burden of CAP in Europe, because precise data are scarce [4]. The highest incidence of CAP occurs among young children and older adults [5], whereas mortality is highest among older adults [6–8]. Many CAP cases require hospitalization and some studies report admission rates between 20 and 30% [9, 10]. Several studies have shown that the incidence rates of hospitalization and the risk of death, vary with age, with the highest rates occurring in the most extreme age groups, mainly over 75 years old [1, 10].

Populations worldwide are expected to age significantly in the coming decades. In Europe, 25% of the population is already aged 60 years or over and that proportion is projected to reach 35% in 2050 and 36% in 2100 [11]. This scenario implies that CAP related outcome may continue to have a high clinical and economic impact by the expected increase of hospital admissions, costs and potentially deaths [9–11].

Despite many studies carried out, in Europe, it remains difficult to understand discrepancies between countries and geographic regions [4, 12–16]. The analyses of data from one or a few hospitals have the potential of introducing bias. This limitation, however, can be overcome by using data from large national database samples, making possible a more robust analysis about the real burden of CAP.

In Portugal, there are some studies that highlight the evolutionary perspective of CAP hospitalizations on mainland hospitals of National Health Service, between 2000 and 2009 [13, 17, 18]. However, in these studies, the phenomenon was explored, a decade ago and only in the adult population.

The present study aimed to characterize the trends of CAP hospitalization episodes in all age groups and to identify the factors associated with their mortality, during a 15-year period, between 2000 and 2014, in Portugal Mainland.

## Methods

### Study design and data collection

A cross-sectional study was conducted using data from 2000 to 2014, on hospitalizations due to CAP. CAP hospitalization was defined as an episode with the principal diagnosis coded as 480–486, from the International Classification of Diseases (ICD), 9th Revision Clinical Modification (ICD-9-CM). Patients, in whom pneumonia was not the main diagnosis, were excluded. Data were obtained from the National Hospital Discharge Database owned and managed by the Central Administration for the Health System of the Portuguese Ministry of Health. This database contains administrative and clinical data of all hospital admissions of the public National Health Service (NHS) in Portugal (up to 2014, it only included Portugal Mainland public hospitals). These data were recorded through the WebGDH computer application (a webpage application with restricted access) and coded according to diagnoses and performed procedures, during the hospital stay, by specially trained health staff using the ICD-9-CM.

Study population encompassed individuals from all age groups, in Portugal Mainland. For each case, information was obtained concerning year of hospital admission, age, sex, unemployment rate of the parish of origin of the patient (value recorded according to the information from the demographic census survey of the Portuguese population [19, 20], chronologically closer to the hospitalization episode), length of stay (LOS), number of procedures performed during hospitalization, and discharge outcome. The anonymity of the subjects was maintained in all the analyzed data, thus informed consent was not required**.** The study was approved by the Ethics Committee of the Lisbon Faculty of Medicine (107/19).

Patients ‘parish unemployment rate was calculated as the number of unemployment persons per 100 active persons registered in the civil parish of origin of the patient, according to census information chronologically closer to the admission episode (2001 Census and 2011 Census) [19, 20].

### Statistical analysis

Sociodemographic and clinical variables were analyzed through descriptive statistics. A comparative analysis was performed to identify associations between variables of interest through Student’s t-test for independent samples in numerical variables or alternatively the non-parametric U Mann-Whitney test. For the comparative analysis of the categorical variables, the Chi-square and the Fisher exact test were used. The statistical analysis assumed a significance level of 5%.

To identify the factors associated with in-hospital mortality a binary logistic regression was used. All variables whose bivariate analysis (Odds Ratio) revealed statistical interest (*p* < 0.20) were included in a multifactorial analysis. The variables in the final model were chosen using the forward conditional method, obtaining the adjusted Odds Ratio.

The quality of the adjustment of the multifactorial model was calculated by the area under the ROC curve (Receiver Operating Characteristic).

To compare the evolution of hospitalization episodes over time, a hospitalization rate per 1000 population, was calculated as the ratio between the number of hospitalization episodes with the main diagnosis of CAP and the number of inhabitants of the same year, multiplied by 1000. The years of 2001 and 2011 were chosen because it was possible to determine accurately the official number of the resident population in Portugal Mainland, based on that year census’ surveys of the Portuguese population [19, 20].

Statistical analysis was performed using the Statistical Package for the Social Sciences (SPSS) version 23 (SPSS Inc., Chicago, IL, USA).

## Results

### Trends in CAP hospitalization rate

Based on data from 2001 and 2011 census, concerning the population resident in Portugal Mainland [19, 20], a CAP population based hospitalization rate was determined, for these two-time points. Across this period, CAP hospitalization rate increased from 2.8 to 4.3 per 1000 population, concerning the overall age groups (Table 1). In both time points, and regarding the studied age groups, hospitalization rates were higher in the extreme age groups ( ≤ 4 and ≥ 75 years). However, when comparing 2001 with 2011, it should be noted that a decrease in CAP hospitalization rate was detected in the age group 0–4 years. In adults, in both time points, the hospitalization rate variation increased progressively with age, with special relevance in the elderly over 75 years, highlighting the observed increase in the absolute number of hospitalizations in 2011, compared to 2001. Between 2001 and 2011, in the age group ≥65 years old, the CAP hospitalization rate increased from 10.3 (range: 5.2–17.6) to 16.5 (range: 6.3–27.6) per 1000 population.

**Table 1:** CAP hospitalization rate based on census population and in-hospital mortality, by age groups, in 2001 and 2011

Age Group	Number of hospitalization episodes		CAP hospitalization rate per 1000 population		Ratio of CAP hospitalization rates	In-hospital mortality		*p*-value*
						% (n)		
	2001	2011	2001	2011	2011/2001	2001	2011	
0–4	3418	2465	6.7	5.4	0.805	0.3% (11)	0.2% (4)	< 0.001
5–14	1163	1097	1.1	1.1	0.964	0.9% (10)	0.4% (4)	0.376
15–24	444	469	0.3	0.4	1.344	5.2% (23)	2.4% (11)	< 0.001
25–44	2013	2036	0.7	0.7	1.029	4.9% (98)	5.6% (113)	0.314
45–64	3562	5393	1.5	2.0	1.331	9.4% (333)	10.5% (568)	< 0.001
65–74	4910	6311	5.2	6.3	1.214	15.2% (746)	15.8% (995)	< 0.001
> 75	11,906	25,571	17.6	27.6	1.562	25.1% (2986)	26.7% (6835)	< 0.001
Total	**27,416**	**43,342**	**2**.**8**	**4**.**3**	**1.550**	**15.4% (4207)**	**19.7% (8530)**	< 0.001

* *p*-value refers to the comparison of in-hospital mortality proportion

### Trends in CAP hospitalizations and in-hospital mortality

**Fig. 1:**
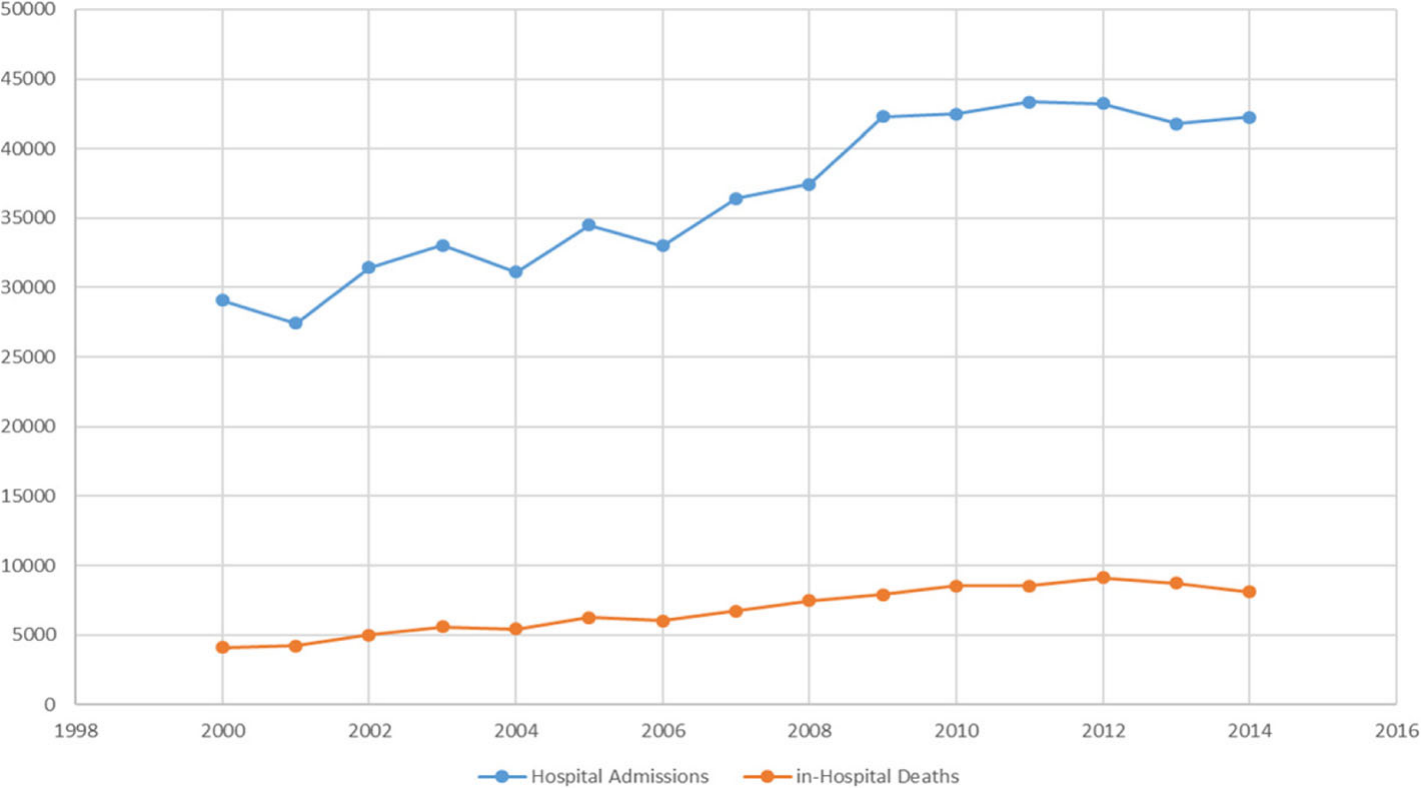
Number of CAP hospital admissions and deaths per year, between 2000 and 2014

**Fig. 2:**
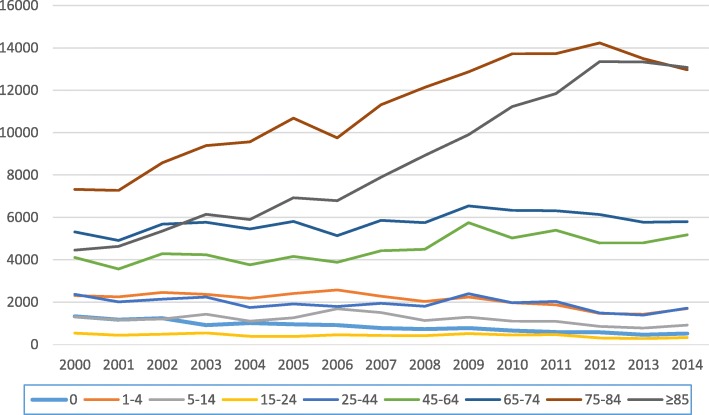
Evolution of the number of CAP hospital admissions by age group, between 2000 and 2014

Between 2000 and 2014, a total of 548,699 hospitalization episodes due to CAP were observed in Portugal Mainland (Figs. 1 and 2). During this time period, there was an increase in the number of annual hospitalization episodes, from 29,065 in 2000 to 42,213 in 2014, corresponding to an overall increase of 45.2%. In the analysis by age group, unlike to the global trend of increased hospitalizations, a pattern of decreased hospitalizations was detected in the group of children under 4 years of age. We also highlight the increase observed in CAP hospitalizations in the age groups over 75 years (Fig. 2). However, since 2012, a slight decrease in the number of hospital admissions was detected in people over 65 years old.

During the 15-year study period, the mean age of CAP hospitalized patients increased from 58.1 ± 29.7 years to 70.7 ± 24.0 years (data not shown), with 70% of hospitalization episodes occurring over 65 years old, mainly in the age group ≥75 years old (Table 2).

**Table 2:** Distribution of hospitalization episodes due to CAP as the main diagnosis (*n* = 548,699) and percentage of in-hospital mortality, according to sociodemographic and clinical variables, between 2000 and 2014

	CAP hospital admission	In-hospital mortality	
	% distribution (n) *or* Mean (± SD) / median	% of Death (n) *or* Mean (± SD) / median	*p*-value *
Age (years)	65.9 (27.0) / 76	80.2 (12.0)/82	< 0.001
Age Group (years)			< 0.001
0–24	12.5 (68486)	0.7% (502)	
25–44	5.3 (28962)	4.9% (1411)	
45–64	12.4 (67870)	9.7% (6568)	
65–74	15.8 (86576)	15.8% (13649)	
75–84	30.4 (167038)	23.1% (38568)	
≥ 85	23.7 (129762)	31.6% (41042)	
Sex			< 0.001
Male	55.1 (302219)	18.9% (57136)	
Female	44.9 (246479)	18.1% (44604)	
Unemployment rate^a^ (%)	10.6 (4.7)/10.4	10.8 (4.6)/10.7	< 0.001
Year of admission			< 0.001
2000–2004	27.7 (152069)	16.0% (24342)	
2005–2009	33.5 (183659)	18.7% (34359)	
2010–2014	38.8 (212971)	20.2% (43039)	
Length of stay (days)	10.6 (11,6)/8	10.0 (15.68)/6	< 0.001
Performed procedures (n)	7.9 (3,7)/8	8.5 (4.01)/8	< 0.001

Legend: * - Mann-Whitney test for numerical variables and Chi-square independence test for categorical variables; ^a^ - Number of unemployment persons per 100 active persons registered in the civil parish of origin of the user, according to census information chronologically closer to the episode (2001 Census and 2011 Census) [19, 20]

**Fig. 3:**
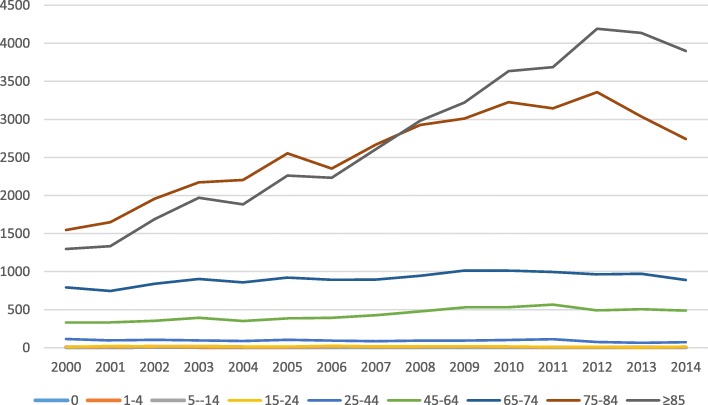
Evolution of the number of in-hospital CAP mortality by age group, between 2000 and 2014

Concerning the absolute number of deaths per year, there was a steady increase until 2012, followed by a decrease in the two consecutive following years (Fig. 1). The analysis by age group showed that, in 2008, the deaths in the population with more than 85 years old exceeded for the first time, the number of deaths in the age group 75–84 years, becoming since then, the age group with the highest number of in-hospital deaths (Fig. 3). Until the end of the period under analysis, despite an observed decrease in the hospital mortality since 2012, the gap between the absolute number of deaths, in the age groups over 85 and 75–84 years, was still increasing, secondary to a higher decrease of deaths in the age group 75–84 years (Fig. 3).

More than a quarter of CAP in-hospital mortality occurred within 72 h. In fact, 10.7% of the mortality was observed within 24 h, 27.5% within 72 h, and 35.9% within 4 days.

Table 2 presents the comparative analysis of in-hospital mortality according to socio-demographic and health variables.

In-hospital mortality, of the overall period under analysis, was 18.5%, with a predominance of males (56.2%) and of the age groups ≥65 years (91.7%).

The number of hospitalized men was almost always higher than that observed in women throughout the life cycle, except in the population over 85 years, where women represented 56.9% (data not shown). Men had a significantly lower mean age (64.3 ± 26.4 years versus 67.9 ± 27.5 years; *p* < 0.001).

Across time, the percentage of deaths increased over each 5 years’ time periods. Deceased patients were significantly older than surviving patients (80.2 versus 65.9 years). Also, deceased patients were submitted to a greater number of procedures and presented a slightly lower LOS (Table 2).

### Factors associated with in-hospital CAP mortality

The sociodemographic and health variables that showed greater association with in-hospital mortality were inserted into a multiple logistic regression analysis model, thus obtaining the magnitude of the association through the determination of Odds Ratio and adjusted Odds Ratio (Table 3).

**Table 3:** Adjusted odds ratio for “death” event according to sociodemographic and clinical variables between 2000 and 2014^a^ (p value < 0.05)

	CAP In-hospital mortality	
	OR (95% CI)	Adjusted OR (95%CI)
Age Group (years)		
< 1	1	1
1–4	0.410 (0.294–0.572)	0.564 (0.404–0,787)
5–14	1.201 (0.892–1.619)	1.697 (1.257–2.290)
15–24	7.149 (5.464–9.354)	9.746 (7.435–12.776)
25–44	9.317 (7.310–11.875)	11.862 (9.286–15.154)
45–64	19.492 (15.364–24.729)	26.098 (20.530–33.175)
65–74	34.050 (26.857–43.169)	47.238 (37.183–60.011)
75–84	54.616 (43.097–69.214)	78.158 (61.545–99.256)
≥ 85	84.156 (66.406–106.651)	124.256 (97.838–157.807)
Sex		
Male	1.055 (1.041–1.070)	1.261 (1.243–1.280)
Female	1	1
Unemployment rate^a^	1.013 (1.011–1.014)	1.005 (1.003–1007)
Year of admission		
2000–2004	1	1
2005–2009	1.208 (1.186–1.230)	1.047 (1.025–1.069)
2010–2014	1.329 (1.306–1.352)	0.961 (0.940–0.982)
Length of stay	0.994 (0.993–0.995)	0.977 (0.976–0.978)
Number of procedures	1.049 (1.047–1.050)	1.035 (1.033–1.037)

Data was also adjusted for Region of Portugal Mainland and for Major Diagnostic Categories (results not presented);

^a^ - Number of unemployment persons per 100 active persons registered in the civil parish of origin of the user, according to census information chronologically closer to the episode (2001 Census and 2011 Census) [19, 20]

According to the adjusted Odds Ratio analysis, the increase in age represented a progressive and significant raise in the probability of death, except for the age group 1–4 years, who showed a lower probability of death. The age group ≥85 years old (Adjusted OR = 124.256; 95%CI: 97.838–157.807) and males (Adjusted OR = 1.261; 95%CI: 1.243–1.280) were significantly associated with death risk for CAP hospitalization. Males had a 26% increased probability of death, compared to females.

The increase of 1 percentage point in the patients ‘parish unemployment rate represented an increase of 0.5% in death probability. Concerning the year of admission, it was observed a decrease in probability of death if the admission occurred in the last quinquennium (2010–2014). After 2010, this risk decreased (Adjusted OR = 0.961; 95%CI: 0.940–0.982).

LOS was associated with the death event and each day of hospitalization represented a decrease of 3.3% in the probability of death. The number of procedures performed during hospitalization was also associated with an increased risk of death, with an increase of 3.5% for each additional procedure.

**Fig. 4:**
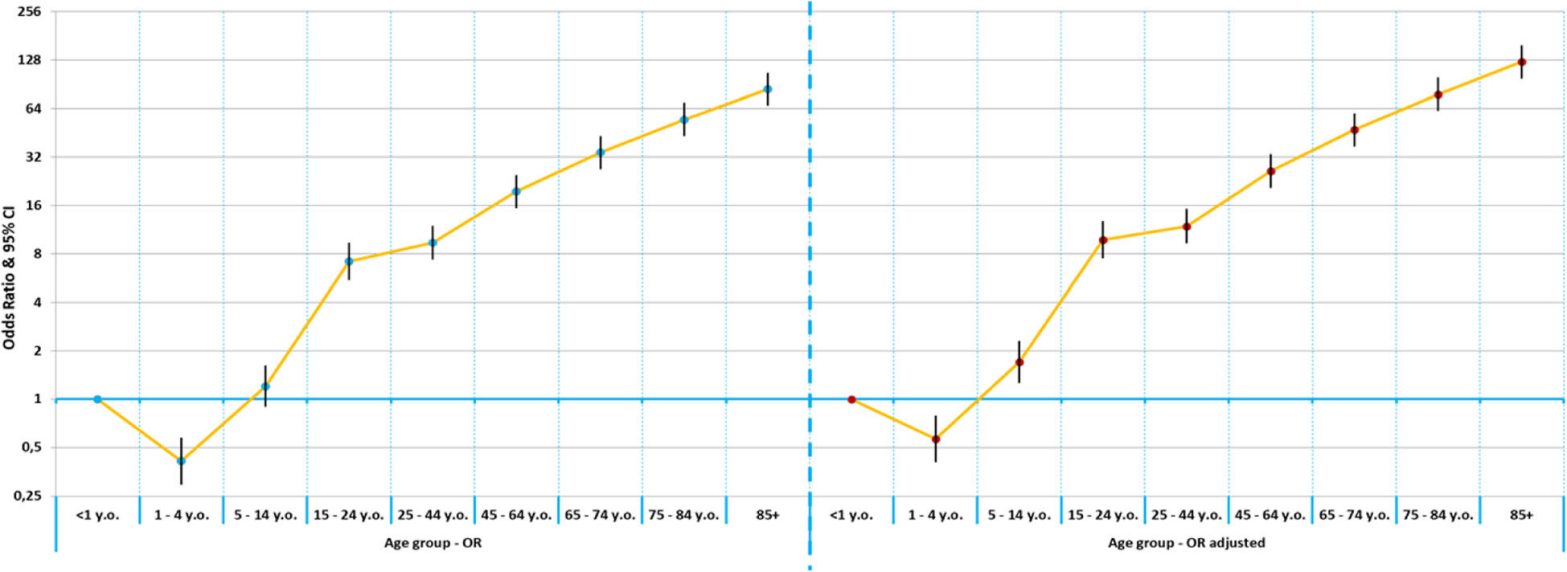
Comparison of Odds Ratio and Adjusted Odd-Ratios for probability of death after hospital admission (age group)

**Fig. 5:**
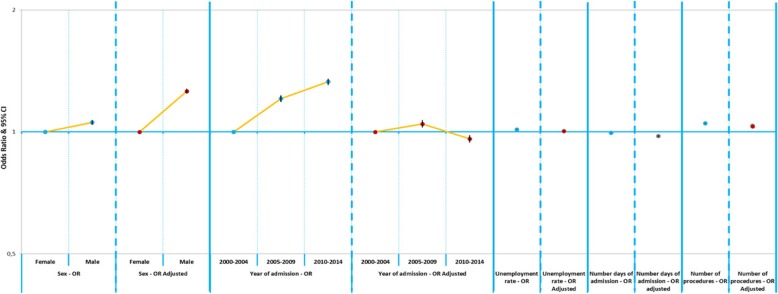
Comparison of Odds Ratio and Adjusted Odd-Ratios for probability of death after hospital admission (sex, year of hospital admission, unemployment rate, number of days of admission, number of procedures)

Figures 4 and 5 represent the comparison of Odds Ratio and Adjusted Odd-Ratios for probability of death after hospital admission (sex, year of hospital admission, unemployment rate, number of days of admission, number of procedures). After the determination of the adjusted Odds Ratio, all variables maintained the same risk trend verified in the analysis of the crude Odds Ratio, except for the quinquennium of admission, since hospitalizations after 2010 showed a lower risk of death.

The determination of the area under the ROC curve resulted in 72.2%, which means a good model was obtained [21].

## Discussion

Our study showed that, in the 15 years under analysis, despite a trend of decrease in CAP hospitalizations and associated mortality after 2012, the number of admissions and in-hospital mortality increased steadily over time. In-hospital CAP mortality was mainly associated with the very old individuals (> 75 years), males, and patients’ parish unemployment rate.

This transversal large-scale study, over a period of 15 years, based on a retrospective data analysis of hospital admission episodes and associated mortality is one of the largest studies of this nature carried out in Portugal, covering all ages, all public hospitals, and the entire mainland population, allowing robust results and comparisons.

Reports on CAP incidence focus mainly on hospitalizations and show wide variability across countries, ranging from 1.48 per 1000 population in England, 2.75 in Germany to 6.27 in Spain [6, 22–24]. Despite our hospitalization rates (2001/2011) are higher than other European countries [25], our data are in line with a previous study in our country [13] and close to reports from Spain [23, 24]. Furthermore, our age group-specific incidence rates increased with advancing age, with incidence rates in older adults very similar to those reported in Spain [24]. This cross-country variability may be related not only to climate differences and vaccine coverage for influenza and pneumococcal vaccines, but also to differences in health system structure and other factors such as heterogeneity in admission criteria [26].

The steady increase of CAP hospitalizations and associated mortality is particularly related to the very old people (Figs. 2 and 3) and reflects most probably the demographic changes that occurred in the Portuguese population during the large study period, with a more aged population according to 2001 and 2011 census data. However, if we look at the numbers associated with the growth of the elderly population and the number of CAP hospitalizations, the increments are disproportionate, taking that in the correspondent period, the growth of population over 75 years was 37.5%, contrasting with an increment of 115% of the hospitalization episodes. This suggests that there are other responsible factors regardless of the change in the population structure. Actually, this disproportionate increase may reflect not only, the weight of ageing, but also the overall impact of the associated comorbidities in the elderly, as documented in several studies [25–28]. Furthermore, apart from the known health consequences of the ageing process, it has been observed that the older Portuguese adults, report a low household income and are a vulnerable group in terms of unhealthy lifestyle behaviors and poor socioeconomic conditions [29]. In particular, older people over 75 years of age who live alone represent a group that is subject to great economic and social vulnerability [30].

During the analyzed period, there was a decrease in hospital admissions and corresponding mortality in the group of children under 4 years, which can be explained by the introduction in Portugal of 7- and 13-valent pneumococcal conjugate vaccine in 2001 and 2009, respectively. Although not included in the National Immunization Program until 2015, their use was widely recommended by pediatricians for all healthy children, allowing increasing vaccination coverages, 33–72% between 2001 and 2013 [31], eventually justifying the reduction of hospital admissions and their mortality taking into account that *Streptococcus pneumoniae* is the main etiological agent of CAP [4].

Regarding the in-hospital mortality associated with CAP, our exploratory model of the factors associated with the probability of death revealed that the increase in age was the factor that contributed most to the rise in death probability, mainly in the very old (> 85 years). In fact, 91.7% of all deaths occurred in the age group ≥65 years old.

The overall mortality rate was 18.5% over the 15 years analyzed. This value is higher than that found in several studies ranging from 4.8 to 14.4% [3, 25, 27, 28], but is similar to other series of various studies conducted in Europe [4]. A possible explanation the observed higher in-hospital mortality rate of CAP may be related to the progressive ageing of Portugal elderly population where very older people (aged 85 and over) are observed to be growing at a faster rate than any other age group. Indeed, Portugal stands out from the rest of the EU countries, as it is one of the fastest ageing countries, considering that by 2050 those aged 55 and over should represent almost half (47.1%) of the total population [32]. In addition, Teixeira-Lopes hypothesized that the fear of dying at home, of patients and family members, could explain the inclusion of many patients with end-of-life pneumonia, especially in older age groups, and we believe this may have impact on observed hospital mortality [33].

In one study where three world regions were analyzed, CAP mortality was 13.3% in Latin America, 9.1% in Europe and 7.3% in US/Canada [34]. After adjustment for confounding variables, estimated differences in mortality between the three regions were significantly reduced. The factors that contributed to the differences found were the incidence of H1N1 infection, comorbidities (cerebrovascular disease), abnormal laboratory values (elevated blood urea nitrogen), antimicrobial therapy (macrolide use, fluoroquinolone use) and vaccinations (influenza, pneumococcal). Some of these factors, may explain the numbers observed in our country, however, given the limitations related to the use of an administrative database we were unable to include accurate data concerning the above-mentioned factors.

After 2012, the overall trend of growth in CAP hospitalizations and associated mortality changed. This change, in particular the decrease in the number of deaths in the last 2 years of the study and a reduction in the risk of death between 2010 and 2014, may be related to the creation and implementation of a national program for chronic respiratory diseases in 2012 [35, 36]. This program under the guidance of the Directorate-General of Health was responsible and encouraged the creation and implementation of respiratory guidelines and health policies in our country. We highlight the free introduction of the influenza vaccine in the population over the age of 65 and in other at-risk groups since the 2012/2013 winter season. In fact, with this public health policy influenza vaccination coverage rate in the population aged 65 and older increased from 43 to 50%, between 2012 and 2014 [37, 38]. Previous studies have documented an association between influenza vaccination and a less severe clinical course of CAP and an improved survival in hospitalized patients with this disease, during influenza season [39, 40]. Moreover, this reduction in CAP hospitalizations associated with the observed risk of death reduction in the five-year period 2011–2014 compared to the previous five-year periods, may also be related to the introduction in Portugal of the 13-valent pneumococcal conjugate vaccine, as referred in the study of Kislaya, where a reduction of pneumococcal pneumonia admissions in Portuguese elderly was attributed to the hypothetical indirect effect (Herd effect) of pneumococcal child vaccination [31].

Our results showed a sex gap, with men presenting higher numbers of CAP hospitalizations, mortality and a higher risk of death during hospitalization. These results are in accordance with other studies that show a sex inequality in health indicators associated with hospitalizations [3, 4, 12, 29], with men presenting consistently higher hospitalization rates for CAP, which may be related to a great number of risk factors, such as smoking, alcoholism, and exposure to toxic occupational exposure [3, 29]. The greater ratio of male to female mortality may also reflect higher rates of smoking in males pre-disposing to respiratory disease [3, 16].

Our study revealed a mean LOS of 10.6 days and a median of 8 days which was in line with other reports [3, 29, 34]. Eventual differences between studies and countries are related to the availability of continuing care outside the hospital environment [41]. In our model for each day of hospitalization, the risk of death decreased 2.3%, which we interpret as a reflex of the interruption of hospitalization by death, given that more than one-third of the deaths (35.9%) occurred within the first 4 days of admission.

There are few reports on the association between the unemployment rate and health status [42]. In our study, patients’ parish unemployment rate was positively associated with CAP mortality. This is the first study that links CAP mortality with unemployment rate as a proxy for the regional economic situation. Given that a higher unemployment rate may generally be related to a more adverse economic condition and this has been associated with increased bacterial pneumonia hospitalization rates [28], our hospital mortality rates may have been inflated by the impact of the economic crisis. This relationship points to the need for specific socioeconomic, regional and population policies in the most vulnerable groups, such as the elderly in social deprivation. It also hints that common spatial processes (environmental-level factors) may be involved in pneumonia outcome, determining patterns and geographic variations and justifying future investigations.

There are limitations to this study. First, it was conducted using retrospective administrative data, conditioning the lack of information about comorbidities, microbiologic etiology, the process of care and influenza/pneumococcal vaccination status. Also, it was not possible to exclude patients who lived in nursing homes or in long-term care institutions in which it was possible, that pneumonia cases were healthcare related. Second, the ICD-9 codes did not allow for the exclusion of nosocomial infections as no code specific to this diagnostic category exists, with the consequent overestimation of CAP hospitalization rates. Third, changes, across the time period in analysis, concerning admission guidelines, diagnosis and coding practices might be probable sources of bias.

## Conclusions

Our study reveals a huge burden of CAP hospitalizations and mortality in the older population, showing that CAP can be a major public health concern, even in a developed country, such as Portugal. In-hospital mortality was particularly related to the ageing process and deprived socioeconomic conditions. Therefore, implementation of CAP preventive measures should be reinforced in these vulnerable groups, concerning smoking cessation, flu, and pneumococcal vaccinations to reduce the burden, particularly in the older population. Also, our findings highlight that even small shifts in unemployment rate are associated with in-hospital CAP mortality, suggesting that unemployment might be an important social determinant.

## Data Availability

The data that support the findings of this study are available from [ACSS] but restrictions apply to the availability of these data, which were used under license for the current study, and so are not publicly available. Data are however available from the authors upon reasonable request and with permission of [ACSS].

## Abbreviations

95% CI: 95% Confidence intervals
ACSS: Central Administration for the Health System
CAP: Community-associated pneumonia
CI: Confidence intervals
EU: European Union
ICD: International Classification of Diseases
LOS: Length of stay
OR: Odds ratio
ROC: Receiver Operating Characteristic
